# New Insights and Evidence on “Food Intolerances”: Non-Celiac Gluten Sensitivity and Nickel Allergic Contact Mucositis

**DOI:** 10.3390/nu15102353

**Published:** 2023-05-17

**Authors:** Nicoletta Greco, Annalinda Pisano, Laura Mezzatesta, Marta Pettinelli, Arianna Meacci, Maria Gemma Pignataro, Carla Giordano, Antonio Picarelli

**Affiliations:** 1 Department of Translational and Precision Medicine, Sapienza University of Rome, 00185 Rome, Italy; nicoletta.greco@uniroma1.it (N.G.); arianna.meacci@gmail.com (A.M.); 2 Department of Radiology, Oncology and Pathology, Sapienza University of Rome, 00185 Rome, Italy; annalinda.pisano@uniroma1.it (A.P.); laura.mezzatesta93@gmail.com (L.M.); mariagemma.pignataro@uniroma1.it (M.G.P.); carla.giordano@uniroma1.it (C.G.)

**Keywords:** irritable bowel syndrome (IBS), celiac disease, food intolerance, nickel allergy, allergic contact dermatitis (ACD), non-celiac gluten sensitivity (NCGS), patch test, intestinal mucosal atrophy, immunohistochemical markers, intestinal vascular reaction

## Abstract

The clinical examination of patients often includes the observation of the existence of a close relationship between the ingestion of certain foods and the appearance of various symptoms. Until now, the occurrence of these events has been loosely defined as food intolerance. Instead, these conditions should be more properly defined as adverse food reactions (AFRs), which can consist of the presentation of a wide variety of symptoms which are commonly identified as irritable bowel syndrome (IBS). In addition, systemic manifestations such as neurological, dermatological, joint, and respiratory disorders may also occur in affected patients. Although the etiology and pathogenesis of some of them are already known, others, such as non-celiac gluten sensitivity and adverse reactions to nickel-containing foods, are not yet fully defined. The study aimed to evaluate the relationship between the ingestion of some foods and the appearance of some symptoms and clinical improvements and detectable immunohistochemical alterations after a specific exclusion diet. One hundred and six consecutive patients suffering from meteorism, dyspepsia, and nausea following the ingestion of foods containing gluten or nickel were subjected to the GSRS questionnaire which was modified according to the “Salerno experts’ criteria”. All patients underwent detection of IgA antibodies to tissue transglutaminase, oral mucosal patch tests with gluten and nickel (OMPT), and EGDS, including biopsies. Our data show that GSRS and OMPT, the use of APERIO CS2 software, and the endothelial marker CD34 could be suggested as useful tools in the diagnostic procedure of these new pathologies. Larger, multi-center clinical trials could be helpful in defining these emerging clinical problems.

## 1. Introduction

It is a common observation that there is a close relationship between the ingestion of certain foods and the appearance of various symptoms. Until now, the occurrence of these events has been generically defined as food intolerance [1,2].

Scientific interest in this topic has been growing in recent years. These conditions should be more properly indicated with the term adverse food reactions (AFRs), which are defined as the appearance of any anomalous clinical reaction following the ingestion, contact, or inhalation of foods or additives contained therein [3]. In this condition, the affected population presents with a wide variety of symptoms that are generally identified as an IBS-like syndrome, including bloating, diarrhea, and abdominal pain [4]. Additionally, various systemic manifestations, such as dermatological, joint, and respiratory disorders, may also occur in affected patients [5]. Furthermore, neurological and behavioural disorders, such as foggy mind, impaired motor coordination, and space–time disorientation can also frequently be observed [6].

Overall, immune-mediated reactions (food allergies), adverse reactions to gluten containing foods (celiac disease, CD; non-celiac gluten sensitivity, NCGS), adverse reactions to nickel containing foods (nickel allergic contact mucositis, Ni-ACM), enzyme deficiencies (lactose intolerance), and disorders related to the ingestion of fermentable oligo-, di- and monosaccharides and polyols (FODMAPS) can be, all together, identified as AFRs [7,8]. Although the etiology and pathogenesis of several AFRs is already known, others, such as NCGS and Ni-ACM, are still not completely defined.

NCGS, also known as “non-celiac gluten sensitivity”, is a clinical condition characterized by intestinal and extra-intestinal symptoms that occur after the ingestion of gluten-containing foods. In NCGS, the symptoms disappear after the withdrawal of gluten from the diet [9]. According to several authors, this gluten-related clinical condition is common in Western countries, and its prevalence is estimated to be between 0.5% and 13% [10]. More recent studies have demonstrated an increased prevalence of NCGS in patients diagnosed with IBS [11,12,13,14].

The diagnosis of NCGS is now based on the use of the “Salerno experts’ criteria”. These specific criteria are based on the quantitative assessment of symptom intensity measured by the Gastrointestinal Symptom Rating Scale [15]. According to the current literature, a double-blind placebo gluten-controlled test or a simple open challenge approach is usually performed to make a correct diagnosis of NCGS [8].

Ni-ACM is a different pathological condition characterized by the appearance of low-grade intestinal inflammation and related symptomatology mediated by a local adaptive response following the ingestion of Ni-containing foods [16].

Nickel is the most studied allergenic agent among metals and is considered a ubiquitous hapten that can be absorbed from the intestine via the respiratory route but also via skin contact. In the Western population, as many as 17% of women and only 3% of men complain of nickel-dependent disorders, which can occur at any age and persist for many years and sometimes for life [17]. Ni is a ubiquitous element and is essential for many microorganisms, plants, and animals [18,19]. However, in predisposed subjects, skin contact or ingestion of foods containing Ni can cause inflammatory and allergic clinical manifestations [20]. Ni-rich foods include tomato, cocoa, beans, mushrooms, corn, soybeans, onion, nuts, canned food, tea, and many others [21,22,23].

Although data for allergic contact dermatitis (ACD) suggest a prevalence of approximately 20%, those for Ni-ACM have not yet been determined. It is possible to hypothesize [17] that in the populations of southern Europe that reactivities to nickel, both cutaneous and systemic, are higher due to the greater use of vegetables and legumes which have a higher nickel content.

The etiopathogenetic mechanisms of NCGS and Ni-ACM, as well as their histopathological features, have not yet been defined, although a recent multicenter study on a huge population has shown subtle histological changes of the intestinal mucosa in NCGS, even at the Marsh 0 stage, such as reduced height of the villi, increased depth of the crypts, changes in the villus/crypt ratio, and focal increased lymphocytic infiltration [24]. Instead, as far as Ni-ACM is concerned, there are still no scientific works documenting this new clinical condition.

In the absence of serological biomarkers, diagnosis is currently based only on the resolution of symptoms after gluten or nickel withdrawal. However, in these conditions, which are still being defined, the exclusion of both celiac disease and wheat allergy is mandatory. Recent studies have suggested an oral mucosa patch test to gluten (GOMPT) and to nickel (Ni-OMPT) as a reliable and rapid tool for diagnosing NCGS and Ni-ACM. The treatment of these two pathological conditions involves a diet with a lower content of gluten or nickel, respectively [8].

The aim of our study was to evaluate the relationship between the ingestion of foods containing gluten or nickel and the appearance of specific symptoms as well as clinical improvements after a gluten free and/or nickel free diet. Histological and immunohistochemical changes of the intestinal mucosa were also examined in a subset of these patients. These clinical and morphological features were then compared to active celiac patients and celiac patients in remission on a gluten-free diet.

## 2. Materials and Methods

### 2.1. Population

One hundred and six patients were referred to our tertiary level celiac center by treating physicians between 2015 and 2019 due to the onset of bloating, dyspepsia, and nausea following the ingestion of foods containing gluten or nickel. Each patient received an information sheet with appropriate explanations about the conduct of the study and was given all the time necessary to decide whether to join. A copy of the information sheet and an informed consent form were signed. All patients underwent clinical evaluation for AFR as follows.

### 2.2. Gastrointestinal Symptom Rating Scale (GSRS) Questionnaire, Modified According to the Salerno Experts’ Criteria

A GSRS questionnaire, modified according to the “Salerno experts’ criteria”, was given to all 106 patients enrolled in the study. Patients were subjected to the questionnaire twice: (i) on their regular diet (Time 1) and (ii) 3 months after a gluten or nickel free diet (Time 2), according to the specific diagnosis. The intensity of the reported symptoms was assessed using a numerical scale from 0 to 10 (Supplementary Table S1). The GSRS questionnaire was considered positive if at least 3 symptoms of the 27 considered had a score ≥5 [4].

A regular diet was considered a diet based on the consumption of 3 meals containing approximately 50% carbohydrates, 20% proteins, and 30% fats, which included a fair amount of vegetables and legumes as required in the classic Mediterranean diet.

### 2.3. Anti-Tissue Transglutaminase Antibody Detection

All patients underwent serum IgA anti tissue transglutaminase (tTG) antibody detection.

According to the manufacturer’s instructions, IgA anti-tTG antibodies were detected in serum samples diluted 1:101 by enzyme-linked immunosorbent assay (ELISA) on microtiter-plate wells coated with recombinant human tTG (QUANTA Lite R h-tTG IgA; INOVA Diagnostics, San Diego, CA; distributed by Instrumentation Laboratory, Milan, Italy). According to the manufacturer’s instructions (negative <4 U/mL; weak positive 4–10 U/mL; and positive >10 U/mL), the antibody level 4 U/mL was used as a cut-off to identify anti-tTG positive results. Data were finally expressed as anti-tTG serum levels/cut-off ratios (absolute numbers) [25].

### 2.4. GOMPT

All patients showing serum IgA tTG within normal limits performed a GOMPT. The GOMPT consisted of the application of a 5 mm filter paper disc on the oral mucosa on which a mixture of Vaseline and 10% gluten was placed (1.2 mg of gluten per patch; SigmaAldrich, St. Louis, MO, USA). The GOMPT was positioned and held in place by a transparent adhesive film (FIRMA, Florence, Italy). Two hours after the administration of GOMPT, the presence of mucosal hyperemia, edema, as well as the appearance of blisters and burning at the application site were evaluated by the same clinician. General reactions, such as diarrhea, bloating, abdominal pain, foggy-mind, itching, headache, and arthralgia have also been reported within 48 h of testing [26].

### 2.5. Ni-OMPT

All patients showing serum IgA tTG within normal limits performed a Ni-OMPT. Ni-OMPT consisted of using a 5 mm filter paper disc saturated with a 5% solution of Ni sulphate in petroleum jelly (0.4 mg of Ni sulphate/8 mg of petroleum jelly) applied to the upper labial mucosa, according to the current literature. Two hours after the administration of Ni-OMPT, the presence of mucosal hyperemia, oedema, as well as the appearance of blisters and burning at the application site were evaluated by the same clinician. General reactions, such as diarrhea, bloating, abdominal pain, foggy-mind, itching, headache, and arthralgia have also been reported within 48 h of testing [4,22].

### 2.6. Histologic and Immunohistochemical Analysis of the Duodenal Mucosa

To complete the diagnostic procedure, all patients were requested to perform an esophagogastroduodenoscopy (EGDS). At least four biopsies from each patient were collected during EGDS, including two in the bulb and two in the second duodenal portion.

Samples were oriented and positioned on blotting paper to guarantee proper histological assessment, fixed in 10% formalin, processed, and embedded in paraffin.

Histologic sections were stained with hematoxylin and eosin (HE) and immuno-stained with monoclonal antibodies against CD3 (1:100), CD4 (1:100), CD8 (1:250) (BioSB, Santa Barbara, CA, USA), CD34 (prediluted antibody), tryptase (1:300) (Dako, Glostrup, Denmark), and CD117 (prediluted antibody, Leica Biosystem, Newcastle).

The histological evaluation of biopsies was performed in accordance with updated guidelines published by the Italian Group of Digestive Disorders (GIPAD) [25].

### 2.7. Morphometric Analysis of the Duodenal Mucosa

All the stained sections were digitalized by APERIO CS2 software (Leica Biosystems). The villous height was measured by selecting only well-oriented villi. A mean of 5 villi for each case was obtained. Qualitative and quantitative information were obtained regarding the inflammatory infiltrate. The number of intra-epithelial CD3 positive T lymphocytes (IEL) was recorded. Lymphocytes were counted per 100 enterocytes covering the villous epithelium of the entire villus, as reported in [27].

The number and distribution of CD3, CD4, and CD8 positive T lymphocytes in the lamina propria (expressed as numbers of lymphocytes/0.2 mm^2^) was obtained, including the presence of the linear disposition of T lymphocytes in the deeper part of the mucosa, which is a feature recently described in NCGS biopsies [24].

Finally, capillary density within the villus axis was quantitatively analyzed on specimens that were immuno-stained with antibodies against the endothelial marker CD34 and expressed as the number of intra-villous capillaries/villus area (μm^2^) × 100. In addition, the ratio between the total area of the lumen of intra-villous capillaries/total villous area × 100 was provided.

### 2.8. Exclusion Criteria

Exclusion criteria were: IgE-mediated food allergies, autoimmune disorders, inflammatory bowel diseases, parasitic diseases, and cancer diseases.

### 2.9. Statistical Analysis

All data are expressed as means ± SEMs. Data were analyzed using T-tests or ANOVA procedures followed by multiple pair-wise comparisons adjusted with Bonferroni corrections. Significance was considered at *p* < 0.05. Numerical estimates were obtained with the GraphPad InStat 6 version (GraphPad, Inc., San Diego, CA, USA).

## 3. Results

### 3.1. Clinical Characteristic of Patients

**Figure 1:**
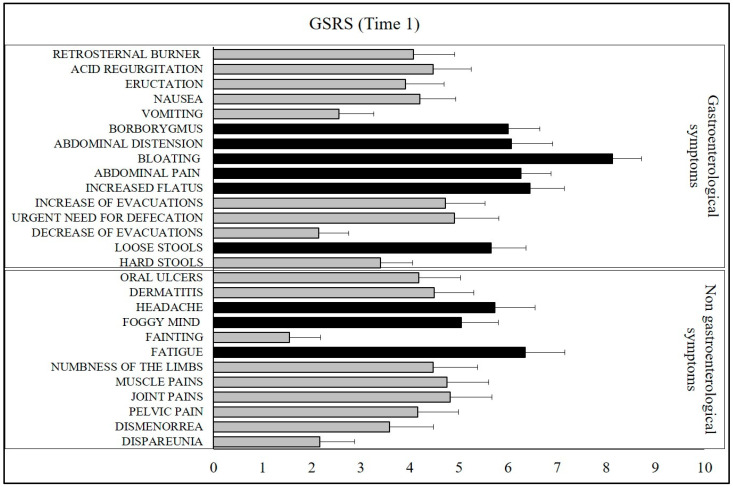
The intensity of symptoms according to the GSRS questionnaire based on the patients’ regular diet (Time 1). The bar graphs represent the mean ± SEM of the GSRS score for each symptom. The black bars indicate the symptoms that exceeded the score ≥5.

All 106 study patients on a free diet (with foods containing gluten and nickel) completed the GSRS questionnaire which was modified according to the Salerno experts’ criteria (Time 1). All study patients, on their regular diet, reported at least 3 symptoms with a score ≥5. On average, the following 9 symptoms scored ≥5: bloating, fatigue, flatulence, headache, abdominal pain, abdominal distension, borborygmus, loose stools, and foggy mind (Figure 1).

Sixty five out of one hundred and six patients showed positive results for serum anti-tTG analysis, suggesting the diagnosis of CD.

The 41 patients who tested negative for serum anti-tTG (39 females, mean age 38 years, range 18–65 years) underwent GOMPT and Ni-OMPT.

Of the 41 patients, 9 (8 females, mean age 43 years, range 27–55 years) showed positive results for GOMPT, suggesting a diagnosis of NCGS (9/106, 8%).

Of the 41 patients, 27 showed positive results for Ni-OMPT (27 females, mean age 37 years, range 25–48 years), suggesting a diagnosis of Ni-ACM (27/106, 25%).

**Figure 2:**
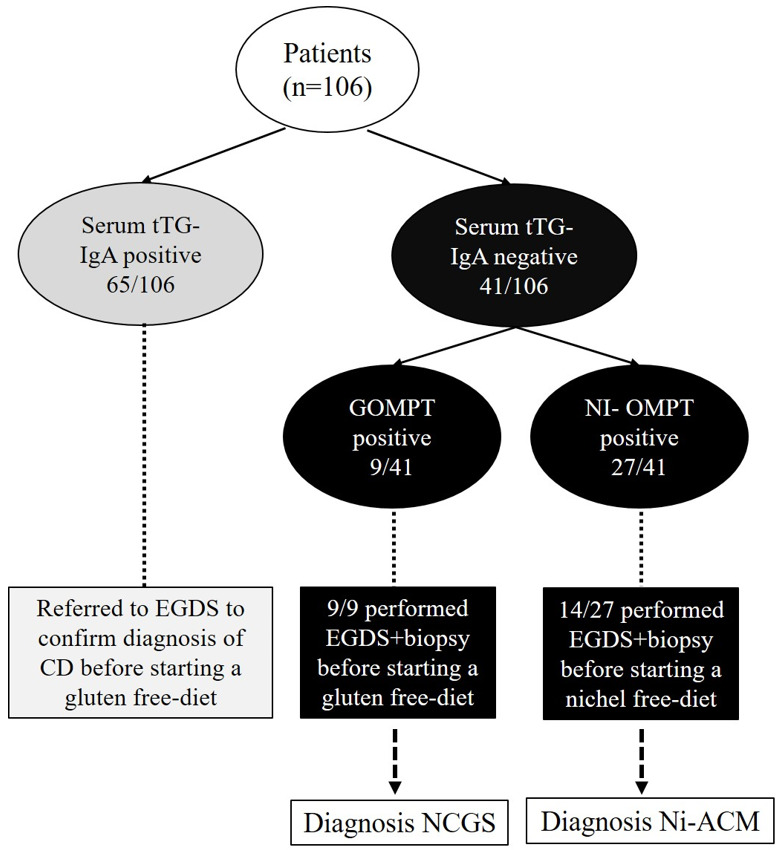
Patients and study design. GOMPT: oral mucosa patch test to gluten; Ni-OMPT: oral mucosa patch test to nickel; NCGS: non-celiac gluten sensitivity; Ni-ACM: nickel allergic contact mucositis.

Finally, of the 41 patients, 5 showed negative results for both GOMPT and Ni-OMPT and were excluded from the study (Figure 2). Patients were then referred to EGDS to complete the diagnostic procedure before starting a specific gluten or nickel-free diet.

### 3.2. GSRS Questionnaire, Modified According to the Salerno Experts’ Criteria in the Three Groups of Patients before and after a Specific Diet

**Figure 3:**
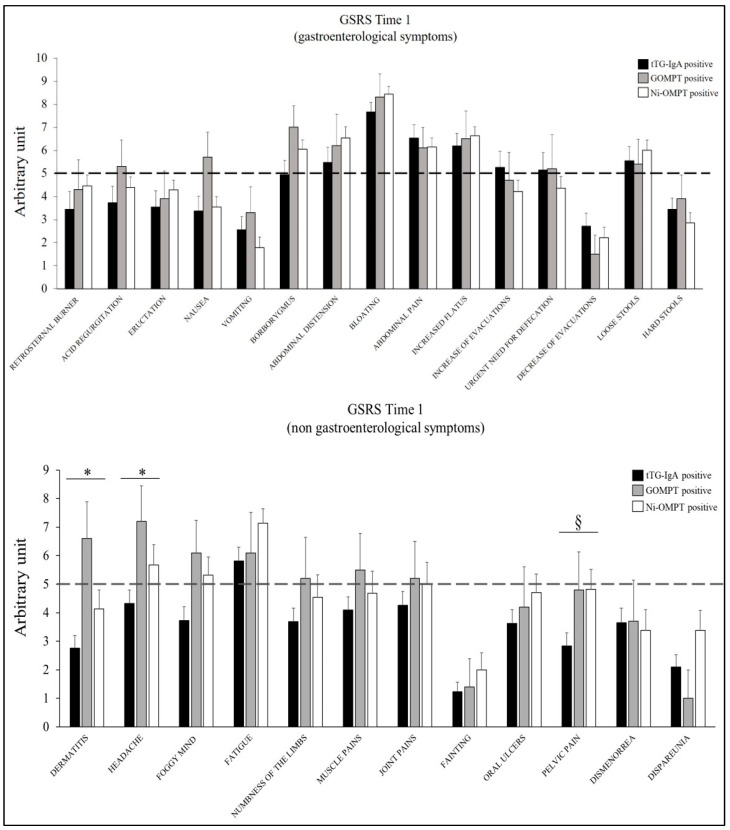
GSRS questionnaire results in anti-tTG-IgA, GOMPT, and Ni-OMPT positive patients on their regular diet (Time 1). The bar graphs represent the mean ±SEM of the GSRS score for each symptom. The GSRS questionnaire is considered positive if at least 3 of the 27 symptoms considered have a score ≥5. The dashed line indicates the cut-off point. GOMPT: oral mucosa patch test to gluten; Ni-OMPT: oral mucosa patch test to nickel. * *p* < 0.05 for GOMPT positive versus anti-tTG-IgA positive patients; § *p* < 0.05 for Ni-OMPT positive patients versus anti-tTG IgA positive patients (ANOVA).

Analysis of the GSRS questionnaire results did not show significant differences in symptom presentation among anti-tTG-IgA, GOMPT, and Ni-OMPT positive patients. Exceptions were dermatitis and headache which showed a statistically significant higher GSRS score in GOMPT positive patients compared to anti-tTG-IgA positive patients. Furthermore, pelvic pain showed a statistically significant higher GSRS score in Ni-OMPT positive patients compared with anti-tTG-IgA positive patients (Figure 3).

After 12 weeks on gluten- or nickel-low diet, patients were required to answer the GSRS questionnaire (Time 2) to evaluate modifications in the severity of symptoms.

**Figure 4:**
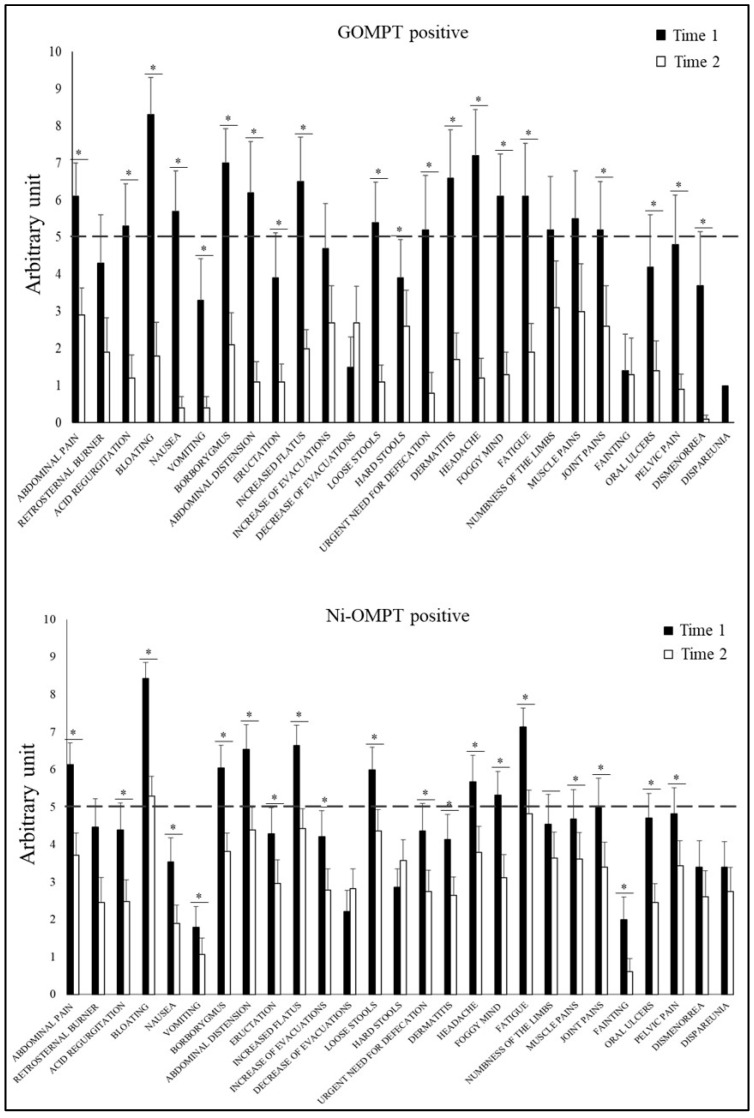
Comparison of GSRS questionnaire results at Time 1 and Time 2 in GOMPT and NI-OMPT positive patients. The bar graphs represent the mean ±SEM of the GSRS score for each symptom. The GSRS questionnaire is considered positive if at least 3 of the 27 symptoms considered have a score ≥5. The dashed line indicates the cut-off point. GOMPT: oral mucosa patch test to gluten; Ni-OMPT: oral mucosa patch test to nickel. * *p*-value < 0.05 for Time 2 (after their specific diet) versus Time 1 (on their regular diet).

All groups showed a significant decrease in the intensity of most symptoms (Figure 4 and Supplementary Figure S1).

### 3.3. Histologic Analysis of the Duodenal Mucosa of GOMPT-and Ni-OMPT Positive Patients

All 9 GOMPT positive patients and 14 of the 27 Ni-OMPT positive patients agreed to perform EGDS before starting the specific diet. None of the biopsies showed features typical of CD at histology (i.e., villi were digitiform, villus/crypt ratio was ≥3, and IEL was <25/100 enterocytes = Marsh 0 stage). No erosion or ulcerations were detected.

Histological data, in association with the mucosal patch test features and the GSRS results after a gluten- or nickel-low diet, corroborated the diagnostic hypothesis of NGCS and Ni-ACM.

**Figure 5:**
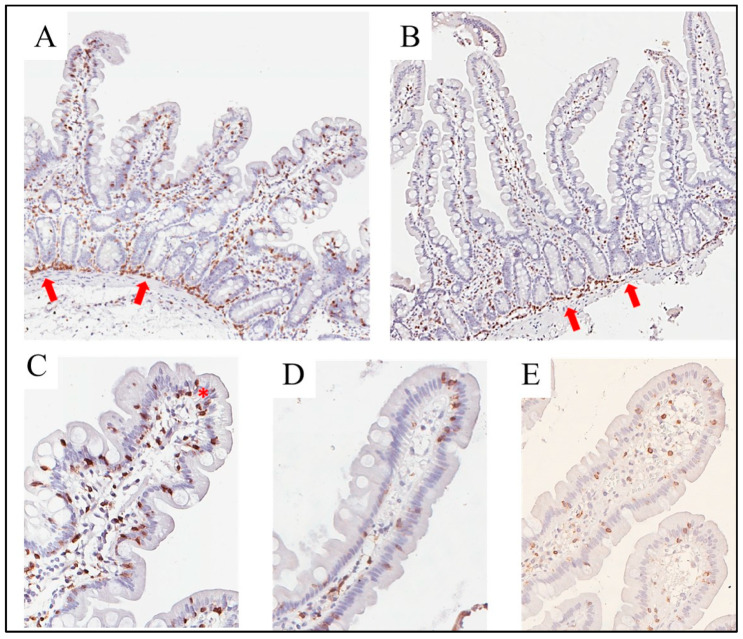
Duodenal biopsies from NCGS (**A**) and Ni-ACM (**B**) immuno-stained with anti-CD3 antibodies. The linear disposition of T lymphocytes in the deeper part of the mucosa is indicated by arrows (original magnification 4×). Higher magnification of a villus from NCGS (**C**), Ni-ACM (**D**), and a control patient who underwent endoscopy for cancer screening (**E**). An asterisk highlights a cluster of intraepithelial T lymphocytes in NCGS (original magnification 20×). NCGS: non-celiac gluten sensitivity; Ni-ACM: nickel allergic contact mucositis.

As shown in Figure 5, all biopsies from NCGS patients showed a linear disposition of T lymphocytes in the deeper part of the mucosa, as previously described in [24]. This finding was also observed in 10 of the 14 (71%) Ni-ACM patients (Figure 5). Small clusters of intraepithelial T lymphocytes, a finding that has been previously described in NCGS [24,25,27], were occasionally observed in our NCGS biopsies.

### 3.4. Morphometric Analysis of Duodenal Mucosa in NCGS, Ni-ACM, Active CD, and CD in Remission versus Controls

To better characterize the morphologic features of NCGS and Ni-ACM, the length of villi was measured. In addition, a quantitative analysis of inflammatory infiltrate was performed on duodenal sections immuno-stained with antibodies against CD3, CD4, and CD8. Findings obtained from the 9 NCGS and 14 Ni-ACM patients were compared to those obtained from 14 selected patients with active CD (CD-Act, positive serum anti-tTG-IgA, and histological damage type 3B-C, according to Marsh–Oberhuber; 6 female, mean age 40 years, range 15–78 years). As controls, we selected two populations: duodenal biopsies obtained from 7 patients with CD in remission who underwent follow-up EGDS during a gluten free diet (CD-Rem, negative serum anti-tTG-IgA, and March 0 at histology; 5 female, mean age 49 years, range 29–79 years) and 11 patients who underwent EGDS because of cancer screening (CTR, 9 female, mean age 46 years, range 23–62 years).

**Figure 6:**
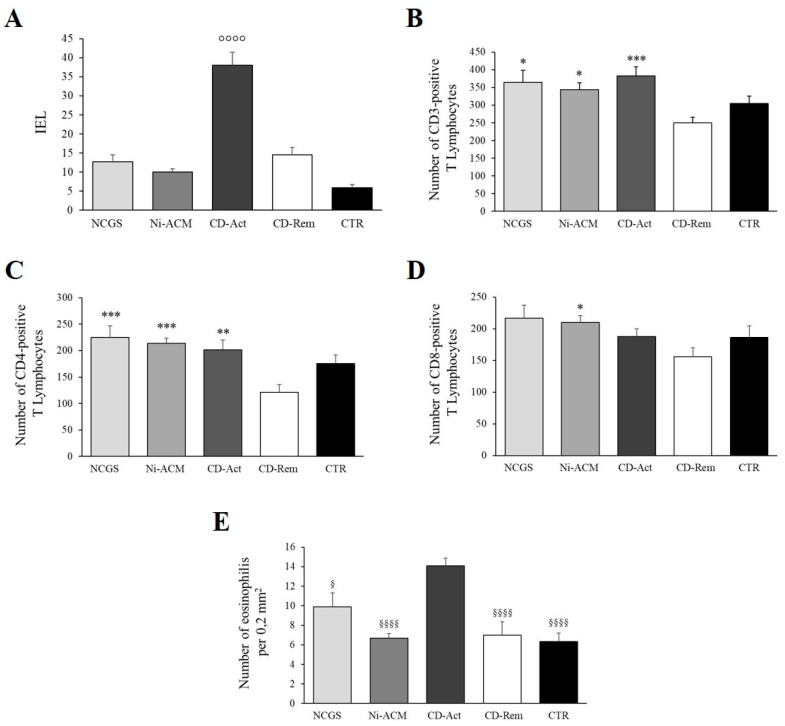
Histomorphometric quantification of IEL (**A**), CD3 (**B**), CD4 (**C**), and CD8 (**D**) positive T lymphocytes and eosinophils (**E**) in the mucosa of duodenal biopsies. IEL: the number of CD3 positive intraepithelial lymphocytes/100 enterocytes; CD-Rem: celiac disease in remission; CD-Act: active celiac disease, NCGS: non-celiac gluten sensitivity; Ni-ACM: nickel allergic contact mucositis. * *p* < 0.05, ** *p* < 0.005, *** *p* < 0.0005 for NCGS, Ni-ACM, and CD-Act versus CD-Rem; § *p* < 0.05 and §§§§ *p* < 0.0001 for all groups versus CD-Act; °°°° *p* < 0.0001 for all groups versus CD-Act (ANOVA).

We did not find significant differences among NCGS, NI-ACM, and CD-Rem versus the controls regarding the IEL, while it was significantly increased in CD-Act compared to all the other groups, as expected (Figure 6A). Instead, when analyzing the number of inflammatory cells within the lamina propria (normalized per 0.2 mm^2^), we observed an increase in CD3 and CD4 positive lymphocytes in NCGS, Ni-ACM, and CD-Act versus CD-Rem and CTR patients, reaching a statistically significant difference only versus CD-Rem (Figure 6B,C). CD8 positive lymphocytes appeared to significantly increase only in Ni-ACM compared to CD-Rem (Figure 6D). The eosinophils amount within the lamina propria appeared to significantly increase in the CD-Act biopsies compared with all the other samples (mean value 13.04/0.2 mm^2^) (Figure 6E). A few mast cells were occasionally demonstrated within the mucosa without significant differences between the four groups (not shown). Finally, we did not find significant differences in the villous length between NCGS and Ni-ACM versus the controls, although NCGS showed a slight, not statistically significant villous length decrease (Supplementary Figure S2).

**Figure 7:**
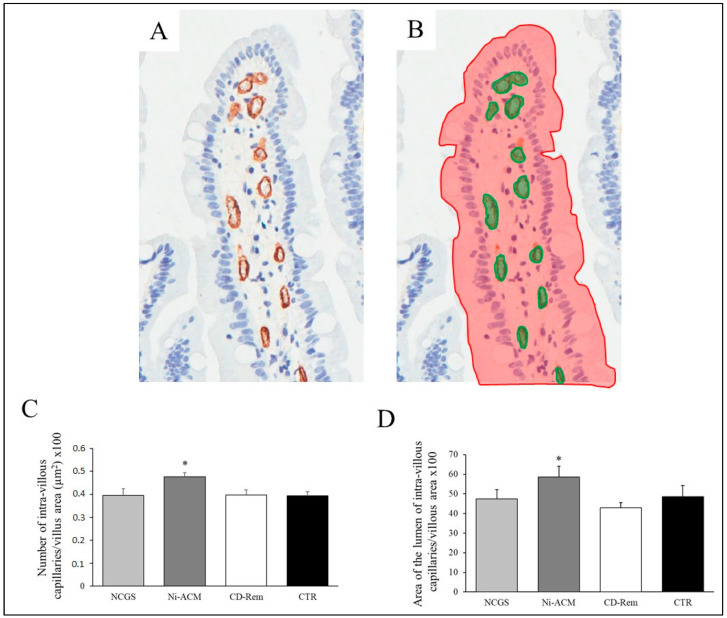
Histomorphometric evaluation of capillary density on duodenal biopsy, including a representative image of a villus immuno-stained with an antibody specific for endothelial cells (CD34) (**A**) and the measurement of capillary (green) and villous (red) areas (**B**). The bar graphs represent the number of intra-villous capillaries/villus area (μm^2^) × 100 (**C**) and the ratio between the total area of the lumen of intra-villous capillaries/total villous area × 100 (**D**). CD-Rem: celiac disease in remission; NCGS: non-celiac gluten sensitivity; Ni-ACM: nickel allergic contact mucositis; CD-Act: active celiac disease; CTR: controls. * *p* < 0.05 for Ni-ACM versus CD-Rem (ANOVA).

To evaluate if a vascular reaction could be involved in the Ni-ACM manifestations, as previously suggested in [28], we evaluated the density of the capillary within the villous axis of NI-ACM, NCGS, and CD-Rem and the controls, showing that the number of capillaries per millimeter was significantly increased only in Ni-ACM versus CD-Rem (Figure 7).

## 4. Discussion

In the present study, we identified 9 patients with suspected NCGS and 27 with Ni-ACM out of a total of 106 patients who were referred to our center because of IBS-like symptoms, primarily bloating.

The diagnosis was obtained by all the patients in the study answering a GSRS questionnaire which was modified according to the Salerno experts’ criteria to which we added, even in the absence of appropriate validation, questions about their gynecological well-being.

The diagnosis was completed using GOMPT and/or Ni-OMPT administered to all patients who tested negative for anti-tTG-IgA serum antibodies. The diagnosis of NCGS was confirmed by the resolution of symptoms after an appropriate privative diet and by the absence of histologic features typical of CD at duodenal biopsy, as previously reported in [15]. Only 14 of the 27 Ni-OMPT positive patients agreed to perform EGDS; thus, in 13 of the 27 patients, a diagnosis of Ni-ACM was based only on the Ni-OMPT results and on the resolution of symptoms after a nickel-low diet. Of note, in the 14 patients for whom biopsies were available, histologic findings were negative for CD.

According to our results, the GSRS questionnaire is a useful tool in combination with GOMPT and Ni-OMPT to identify patients with NCGS and Ni-ACM and to verify symptom changes after an appropriate diet.

In our study, the clinical presentations of NCGS and Ni-ACM were indistinguishable from those of CD. Exceptions were the higher severity of dermatitis and headache in NCGS patients and pelvic pain in Ni-ACM patients.

An interesting finding in our study is the demonstration of low-grade inflammation in the duodenal mucosa of both NCGS and Ni-ACM patients, as highlighted by the increase in CD3, CD4, and CD8 T lymphocytes compared with the controls (both CTR and CD-Rem). This increase was statistically significant only versus CD-Rem. The latter finding could be explained by the low number of observations and by the intrinsic variability of the CTR group, which was characterized by an adult population with an uncontrolled and heterogeneous diet, compared with the more uniform CD-Rem group, which was composed of subjects on gluten-free diets with no other gastrointestinal disease. We did not find differences in the IEL amount and villous height in Ni-ACM and NCGS compared to the controls (both CTR and CD-Rem). Overall, our results are in line with recent findings highlighted by a multicenter study on a large number of patients (175) [24] and confirm that minimal morphological alterations and slight inflammation characterize the duodenum of patients affected by NCGS. The small number of patients involved in our study probably prevented us from demonstrating a slight increase in IEL and/or a decrease in villous height compared with CTR, as previously shown in [24]. In Ni-ACM patients, the observed increase in lamina propria inflammation was associated with a significant increase in vascular density and total lumen vessels area in the villous axes, consistent with a local vascular reaction. In our previous work, we have already shown that an increase in CD3+ cells can be observed in the oral mucosa of nickel-sensitive patients following Ni-OMPT. In addition, increases in CD4+ and CD8+ cells were observed in the intermediate layers of the oral mucosa and in the capillaries, respectively. The perivascular localization of CD8+ cells shows that the reactions observed after Ni-OMPT are predominantly of the vascular type [29,30]. Therefore, the need to conduct further studies on a larger population to justify the role and importance of the vascular component in nickel-induced inflammatory processes is mandatory.

The clinical improvements obtained from the patients in this study cannot be considered conclusive because, firstly, a larger population would be needed, and secondly, other studies should provide the use of specific rechallenges to confirm the initial data. In summary, our observations point to a direct responsibility of gluten and nickel in the onset of symptoms in NCGS and Ni-ACM patients, respectively. Moreover, we showed low-grade inflammation in duodenal mucosa of both conditions associated in Ni-ACM with a local vascular reaction.

## Data Availability

The data that support the findings of this study are available on request from the corresponding author.

## Supplementary Materials

The following supporting information can be downloaded at: https://www.mdpi.com/article/10.3390/nu15102353/s1, Figure S1: Comparison of GSRS questionnaire results at Time 1 and Time 2 in tTG-IgA positive patients. The bar graphs represent the mean ± SEM of the GSRS score for each symptom. The GSRS questionnaire is considered positive if at least 3 of the 27 symptoms considered have a score ≥5. The red line indicates the cut-off point. * *p*-value < 0.05 for Time 2 (after their specific diet) versus Time 1 (on their regular diet) (T-Test); Figure S2: Histomorphometric evaluation of villous lengths. The bar graphs represent the villous length in µm. NCGS: non-celiac gluten sensitivity; Ni-ACM: nickel allergic contact mucositis; CD-Rem: celiac disease in remission; CTR: controls. Table S1: Gastrointestinal Symptom Rating Scale (GSRS) questionnaire, modified according to the Salerno Experts’ Criteria.
