# Supplementary material for: New Insights and Evidence on “Food Intolerances”: Non-Celiac Gluten Sensitivity and Nickel Allergic Contact Mucositis
Source: Nutrients. 2023 May 17;15(10):2353. doi: 10.3390/nu15102353 (PMC10222428; doi:10.3390/nu15102353)
Supplement: Supplementary file 1 [file nutrients-15-02353-s001.zip › nutrients-2373986-supplementary.pdf]

Supplemental Table S1: Gastrointestinal Symptom Rating Scale (GSRS) questionnaire, modified according to the Salerno Experts' Criteria

ABDOMINAL PAIN	0	1	2	3	4	5	6	7	8	9	10
RETROSTERNAL BURNER	0	1	2	3	4	5	6	7	8	9	10
ACID REGURGITATION	0	1	2	3	4	5	6	7	8	9	10
BLOATING	0	1	2	3	4	5	6	7	8	9	10
NAUSEA	0	1	2	3	4	5	6	7	8	9	10
VOMITING	0	1	2	3	4	5	6	7	8	9	10
BORBORYGMUS	0	1	2	3	4	5	6	7	8	9	10
ABDOMINAL DISTENSION	0	1	2	3	4	5	6	7	8	9	10
ERUCTATION	0	1	2	3	4	5	6	7	8	9	10
INCREASED FLATUS	0	1	2	3	4	5	6	7	8	9	10
INCREASE OF EVACUATIONS	0	1	2	3	4	5	6	7	8	9	10
DECREASE OF EVACUATIONS	0	1	2	3	4	5	6	7	8	9	10
LOOSE STOOLS	0	1	2	3	4	5	6	7	8	9	10
HARD STOOLS	0	1	2	3	4	5	6	7	8	9	10
URGENT NEED FOR DEFECATION	0	1	2	3	4	5	6	7	8	9	10
DERMATITIS	0	1	2	3	4	5	6	7	8	9	10
HEADACHE	0	1	2	3	4	5	6	7	8	9	10
FOGGY MIND	0	1	2	3	4	5	6	7	8	9	10
FATIGUE	0	1	2	3	4	5	6	7	8	9	10
NUMBNESS OF THE LIMBS	0	1	2	3	4	5	6	7	8	9	10
MUSCLE PAINS	0	1	2	3	4	5	6	7	8	9	10
JOINT PAINS	0	1	2	3	4	5	6	7	8	9	10
FAINTING	0	1	2	3	4	5	6	7	8	9	10
ORAL ULCERS	0	1	2	3	4	5	6	7	8	9	10
PELVIC PAIN	0	1	2	3	4	5	6	7	8	9	10
DISMENORREA	0	1	2	3	4	5	6	7	8	9	10
DISPAREUNIA	0	1	2	3	4	5	6	7	8	9	10
OTHER:	0	1	2	3	4	5	6	7	8	9	10

NB: mark with an X the box corresponding to the intensity of the symptom (min = 0, max = 10)

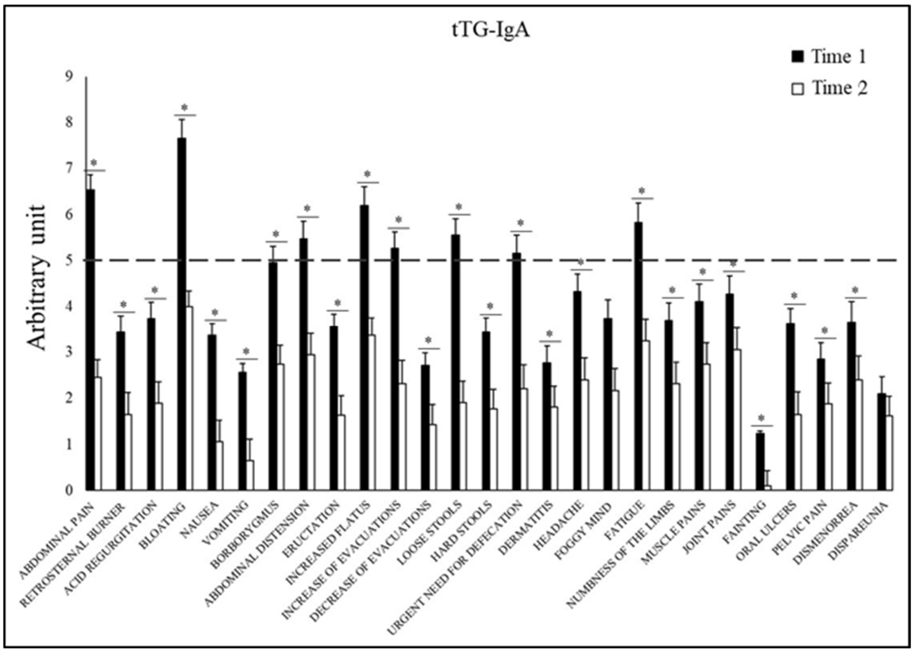

Supplemental Figure S1. Comparison of GSRS questionnaire results at Time 1 and Time 2 in tTG-IgA positive patients. The bar graphs represent the mean ± SEM of the GSRS score for each symptom. The GSRS questionnaire is considered positive if at least 3 of the 27 symptoms considered have a score ≥5. The red line indicates the cut-off point. * p-value < 0.05 for Time 2 (after their specific diet) versus Time 1 (on their regular diet) (T-Test).

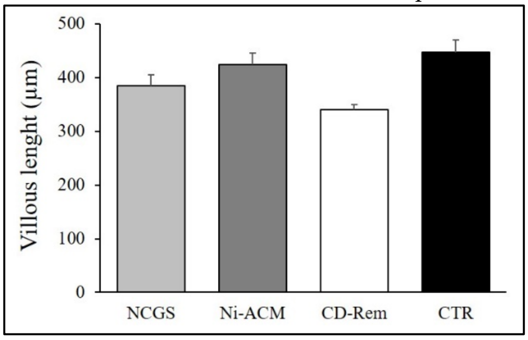

Supplemental Figure S2. Histomorphometric evaluation of villous lengths. The bar graphs represent the villous length in µm. NCGS: non-celiac gluten sensitivity; Ni-ACM: nickel allergic contact mucositis; CD-Rem: celiac disease in remission; CTR: controls.

#

#
